# The expression of chondrogenesis-related and arthritis-related genes in human ONFH cartilage with different Ficat stages

**DOI:** 10.7717/peerj.6306

**Published:** 2019-01-16

**Authors:** Gaoyang Chen, Lei Zhong, Qingyu Wang, Zhaoyan Li, Jing Shang, Qiwei Yang, Zhenwu Du, Jincheng Wang, Yang Song, Guizhen Zhang

**Affiliations:** 1 Department of Orthopedics, The Second Hospital of Jilin University, Changchun, Jilin, China; 2 Research Centre, Second Hospital of Jilin University, Changchun, Jilin, China; 3 The Engineering Research Centre of Molecular Diagnosis and Cell Treatment for Metabolic Bone Diseases of Jilin Province, Changchun, Jilin, China

**Keywords:** Cartilage, ONFH, Gene expression, Different ficat stages

## Abstract

**Background:**

It has been well known that the degeneration of hip articular cartilage with osteonecrosis of the femoral head (ONFH) increases the instability of hip and accelerates the development process of ONFH. A better understanding of the expression of chondrogenesis-related and arthritis-related genes of cartilage along with the progression of ONFH seems to be essential for further insight into the molecular mechanisms of ONFH pathogenesis.

**Methods:**

We analyzed the differentially expressed gene profile (GSE74089) of human hip articular cartilage with ONFH. The functions and pathway enrichments of differentially expressed genes (DEGs) were analyzed via GO and KEGG analysis. The expression of six selected critical chondrogenesis-related and four arthritis-related genes in eight human hip articular cartilage with femoral neck fracture (FNF) and 26 human hip articular cartilage with different stages ONFH (6 cases of Ficat stage II, 10 cases of Ficat stage III and 10 cases of Ficat stage IV) were detected.

**Results:**

A total of 2,174 DEGs, including 1,482 up-regulated and 692 down-regulated ones, were obtained in the ONFH cartilage specimens compared to the control group. The GO and KEGG enrichment analysis indicated that the function of these DEGs mainly enriched in extracellular matrix, angiogenesis, antigen processing and presentation. The results showed a significant stepwise up-expression of chondrogenesis-related genes, including MMP13, ASPN, COL1A1, OGN, COL2A1 and BMP2, along with the progression of ONFH. The arthritis-related genes IL1*β*, IL6 and TNF*α* were only found up-expressed in Ficat IV stage which indicated that the arthritis-related molecular changes were not significant in the progression of ONFH before Ficat III stage. However, the arthritis-related gene PTGS2 was significant stepwise up-expression along with the progression of ONFH which makes it to be a sensitive arthritis-related biomarker of ONFH.

**Conclusion:**

Expression changes of six chondrogenesis-related and four arthritis-related genes were found in hip articular cartilage specimens with different ONFH Ficat stages. These findings are expected to a get a further insight into the molecular mechanisms of ONFH progression.

## Introduction

Osteonecrosis of the femoral head (ONFH) is a crippling disease which was caused by multiple etiologies (Qin et al., 2015). It has an incidence estimated at 150,000 to 200,000 new cases in China (Song et al., 2017) and 20,000 new cases in the United States per year (Seamon et al., 2012). However, the molecular etiology and pathogenesis of ONFH still remain elusive, which impedes the development of effective precautions in its earlier stages and leaves a large number of ONFH patients to total hip replacement. Potential causes of ONFH include trauma, steroids, alcoholism, irradiation and so on (Larson et al., 2018). These risk factors could lead to ischemic changes and eventually result in femoral head collapse and secondary arthritis (Chen et al., 2017). In the early stages of ONFH, the injured blood supply leads to local osteonecrosis and osteoclast-mediated bone resorption (Gangji et al., 2018). Then the osteoblast-mediated reparative reaction initiated (Chen, Liu & Cheng, 2018; Seamon et al., 2012). However, the imbalance between bone reformation and resorption lead to structural damage and collapse of the femoral head (Liu et al., 2016). Cartilaginous tissues of the collapsed femoral head are eventually degenerated and softened due to the reduction of nutritional support from subchondral bone (Madry, Orth & Cucchiarini, 2016; Park et al., 2017). The degenerated articular cartilage could increase the instability of hip which accelerates the development process of ONFH (Hawellek et al., 2018).

With the developing of microarray and sequencing technology, many attempts have been made to reveal the expression profile of RNAs of bone and bone marrow in ischemic femoral head (Chen et al., 2015; Lin & Lin, 2017; Wang et al., 2018). In the past few years, some studies focused on the gene expression profiling of hip articular cartilage from ONFH cases in order to reveal the potential mechanisms of damage to articular cartilage. Liu et al. conducted a genome-wide gene expression profiling of hip articular cartilage with ONFH (GSE74089) and identified 27 differently expressed genes in ONFH articular cartilage (Liu et al., 2016; Wang et al., 2016). However, to date and the best of our knowledge, there is no study has been done to analyze the differential expression of critical chondrogenesis-related and arthritis-related in human ONFH cartilage with different stages.

In this study, based on the dataset GSE74089 from the Gene Expression Omnibus (GEO) database of the National Center of Biotechnology Information (NCBI, http://www.ncbi.nlm.nih.gov/geo/), we analyzed the differentially expressed genes (DEGs) via bioinformatics methods and selected six chondrogenesis-related DEGs and four arthritis-related genes. Then these ten DEGs were validated by 8 human hip articular cartilage with femoral neck fracture (FNF) and 26 human hip articular cartilage with different stages ONFH. Therefore, the aim of our study is to explore the expression changes of critical chondrogenesis-related DEGs and arthritis-related genes in human ONFH cartilage with different stages, which may contribute to a better understanding of the molecular mechanisms of ONFH pathogenesis.

## Materials and Methods

### Microarray data and DEGs

The date of gene expression microarray GSE74089 (GPL13497, Agilent-026652 Whole Human Genome Microarray 4x44K v2; Agilent Technologies Inc., California, USA) was downloaded from GEO datasets. The tested specimens of this gene expression profile contained four hip cartilage samples of ONFH and 4 hip cartilage samples of healthy control. The GEO2R (https://www.ncbi.nlm.nih.gov/geo/geo2r/), an interactive online tool, was applied to analyze the DEGs of ONFH cartilage samples. The group names (test group and control group) were entered after ’Define groups’ was clicked. ONFH cartilage samples were assigned to test group and FHF cartilage samples were assigned to control group. Then the adjustment was applied to the P-values (Benjamini & Hochberg). The adjusted *P* < 0.05 and |LogFC| ≥ 2 were set as the cut off criterions. Then a volcano map was generated according to these criterions.

### GO and KEGG analysis

Gene Ontology (GO) and Kyoto Encyclopedia of Genes and Genomes (KEGG) enrichment analysis were performed via using The Database for Annotation, Visualization and Integrated Discovery (DAVID, https://david.ncifcrf.gov/) tool (Dennis et al., 2003), an online bioinformatics tool which provides a functional interpretation of large lists of genes or proteins. The up-regulated DEGs and down-regulated DEGs were analyzed separately in order to get a better understanding of potential functions of DEGs in ONFH cartilage.

### ONFH and FHF articular cartilage specimens

**Figure 1:**
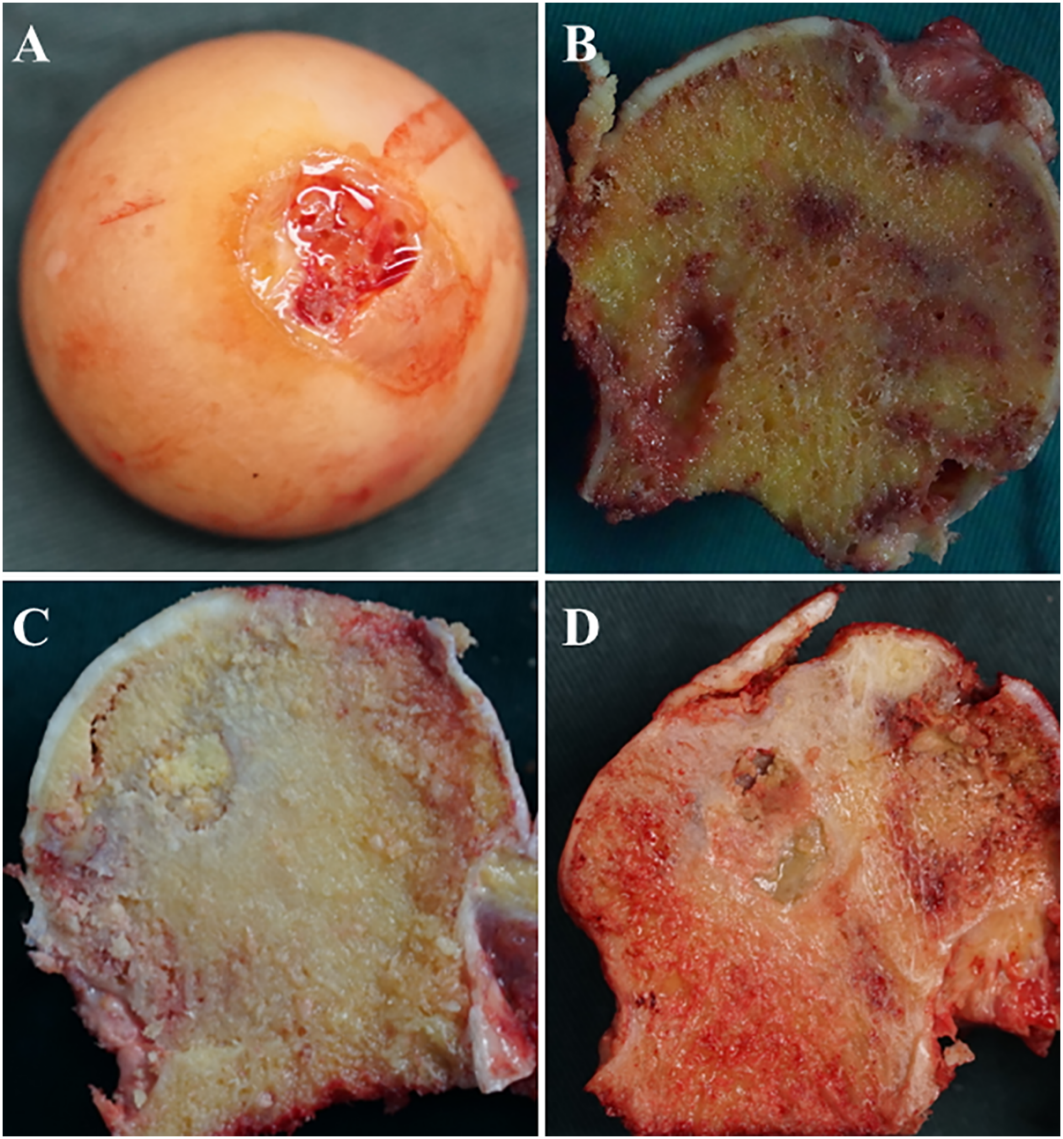
Images of femoral heads and position of specimens. (A) from patient with FHF; (B) from patient with ONFH Ficat stage II; (C) from patient with ONFH Ficat stage III; (D) from patient with ONFH Ficat stage IV.

A total of 8 hip articular cartilage specimens were collected from FHF patients as control subjects. The ONFH hip articular cartilage specimens were collected from 26 ONFH patients who were undergoing total hip replacement at the Second Hospital of Jilin University. According to the Ficat classification (Sultan et al., 2018), these 26 ONFH hip articular cartilage specimens were divided into three groups (6 cases Ficat stage II, 10 cases Ficat stage III and 10 cases Ficat stage IV) as shown in Table 1. Patients concurrent with severe chronic diseases, such as congenital diseases, cardiovascular diseases, diabetes mellitus, renal dysfunction, HIV infection, and cancer, were excluded. All cartilage specimens were sliced to 3 mm depth by a fresh scalpel blade during the operation (Fig. 1). This study was approved by the ethics committee of the Second Clinical College of Jilin University (NO. 2018-082) and all participants have been provided informed consent for their taking part in the study.

**Table 1:** Characteristics of study subjects.

	Number of subjects	Age (years, mean ± SD)	Sex	
			Male	Female
Control	8	74.13 ± 5.49	2	6
Ficat II	6	58.5 ± 10.61	5	1
Ficat III	10	59.14 ± 11.94	9	1
Ficat IV	10	58.75 ± 3.30	7	3

### RNA preparation

Total RNAs of all cartilage specimens were extracted using TRIzol (Invitrogen) following the manufacturer’s introductions. Then RNA concentration was measured by Agilent ND-1000. The cDNAs were synthesized by reverse transcription from total RNA using oligo(dT) primer method (PrimeScriptTMRT Reagent Kit; TaKaRa, Kusatsu, Japan).

### qRT-PCR validation of chondrogenesis-related and arthritis-related genes

A total of 6 chondrogenesis-related genes were selected for qRT-PCR validation, including matrix metallopeptidase 13 (MMP13), asporin (ASPN), collagen type I alpha 1 chain (COL1A1), osteoglycin (OGN), collagen type II alpha 1 chain (COL2A1) and bone morphogenetic protein 2 (BMP2) (Aref-Eshghi et al., 2015; Chou et al., 2013; Ollitrault et al., 2015; Tarpey et al., 2013; Yang et al., 2018), based on results from previous studies and gene function. 4 arthritis-related genes, including interleukin 1 beta (IL1*β*), interleukin 6 (IL6), tumor necrosis factor alpha (TNF*α*) and prostaglandin-endoperoxide synthase 2 (PTGS2) (Chen, Liu & Cheng, 2018), were also selected (Table 2). Quantitative real-time PCR was performed using SYBR Green qPCR Master Mix (Thermo Fisher Scientific). The expression of each mRNA was normalized relative to the endogenous control of human glyceraldehyde-3-phosphate dehydrogenase (GAPDH). The relative expression was calculated using the 2^−ΔΔCt^ method. All the primers were shown in Table 3.

**Table 2:** Selected chondrogenesis-related and arthritis-related genes.

Official symbols	Gene names	Functions	Fold Change (logFC)
MMP13	matrix metallopeptidase 13	Extracellular matrix	6.54
COL1A1	collagen type I alpha 1 chain	Extracellular matrix	5.77
ASPN	asporin	Extracellular matrix	4.33
OGN	osteoglycin	Extracellular matrix	3.62
COL2A1	collagen type II alpha 1 chain	Extracellular matrix	2.35
BMP2	bone morphogenetic protein 2	Cartilage development	2.64
IL1*β*	interleukin 1 beta	Inflammation	−0.13
IL6	interleukin 6	Inflammation	−1.01
TNF*α*	tumor necrosis factor alpha	Inflammation	−1.51
PTGS2	prostaglandin-endoperoxide synthase 2	Inflammation and mitogenesis	3.64

**Table 3:** The primers of mRNAs.

Gene	**Primer sequences**	
MMP13	F: 5′-AGCTGGACTCATTGTCGGGC-3′	R: 5′-AGGTAGCGCTCTGCAAACTGG-3′
COL1A1	F: 5′-GGGATTCCCTGGACCTAAAG-3′	R: 5′-GGAACACCTCGCTCTCCAG-3′
ASPN	F: 5′-TTGAAGGGGTGACGGTGTTC-3′	R: 5′-AGTGAAGCTCCAATAAAGTTGGT-3′
OGN	F: 5′-GCTGAAATGGAGACTGTGCACTCTA-3′	R: 5′-GTTAGAAGTATGACCCTATGGGTA-3′
COL2A1	F: 5′-GTGTCAGGGCCAGGATGT-3′	R: 5′-TCCCAGTGTCACAGACACAGAT-3′
BMP2	F: 5′-TGAGGATTAGCAGGTCTTTG-3′	R: 5′-CACAACCATGTCCTGATAAT-3′
IL1*β*	F: 5′-TGAGCTCGCCAGTGAAATGA-3′	R: 5′-AACACGCAGGACAGGTACAG-3′
IL6	F: 5′-CTCAATATTAGAGTCTCAACCCCCA-3′	R: 5′-GAGAAGGCAACTGGACCGAA-3′
TNF*α*	F: 5′-CTGGGCAGGTCTACTTTGGG-3′	R: 5′-CTGGAGGCCCCAGTTTGAAT-3′
PTGS2	F: 5′-GTTCCACCCGCAGTACAGAA-3′	R: 5′-AGGGCTTCAGCATAAAGCGT-3′
GAPDH	F: 5′-CGGACCAATACGACCAAATCCG-3′	R: 5′-AGCCACATCGCTCAGACACC-3′

### The protein-protein interaction (PPI) networks

GeneMANIA (http://www.genemania.org/) is a web-based database and a tool for the prediction of gene functions on the basis of multiple networks derived from different genomic or proteomic data sources (Franz et al., 2018). It is fast enough to predict gene functions with great accuracy by using GeneMANIA because hundreds of datasets from I2D, BioGRID, GEO and Pathway Commons, as well as organism-specific functional genomics data sets, have been collected in this software. A protein-protein interaction (PPI) network of above chondrogenesis-related and arthritis-related genes was generated using GeneMANIA.

### Statistical analysis

The adjusted *P* < 0.05 and |LogFC| ≥ 2 were set as the cut off criterions for DEGs. The bar graphs of GO enrichment were constructed by Microsoft Excel software and *P* < 0.05 indicated that the difference was statistically significant. The volcano map and frequency distribution maps of DEGs were conducted using GraphPad Prism 7.0 (GraphPad Software Inc., La Jolla, CA, USA).

## Results

### Differentially expressed genes

**Figure 2:**
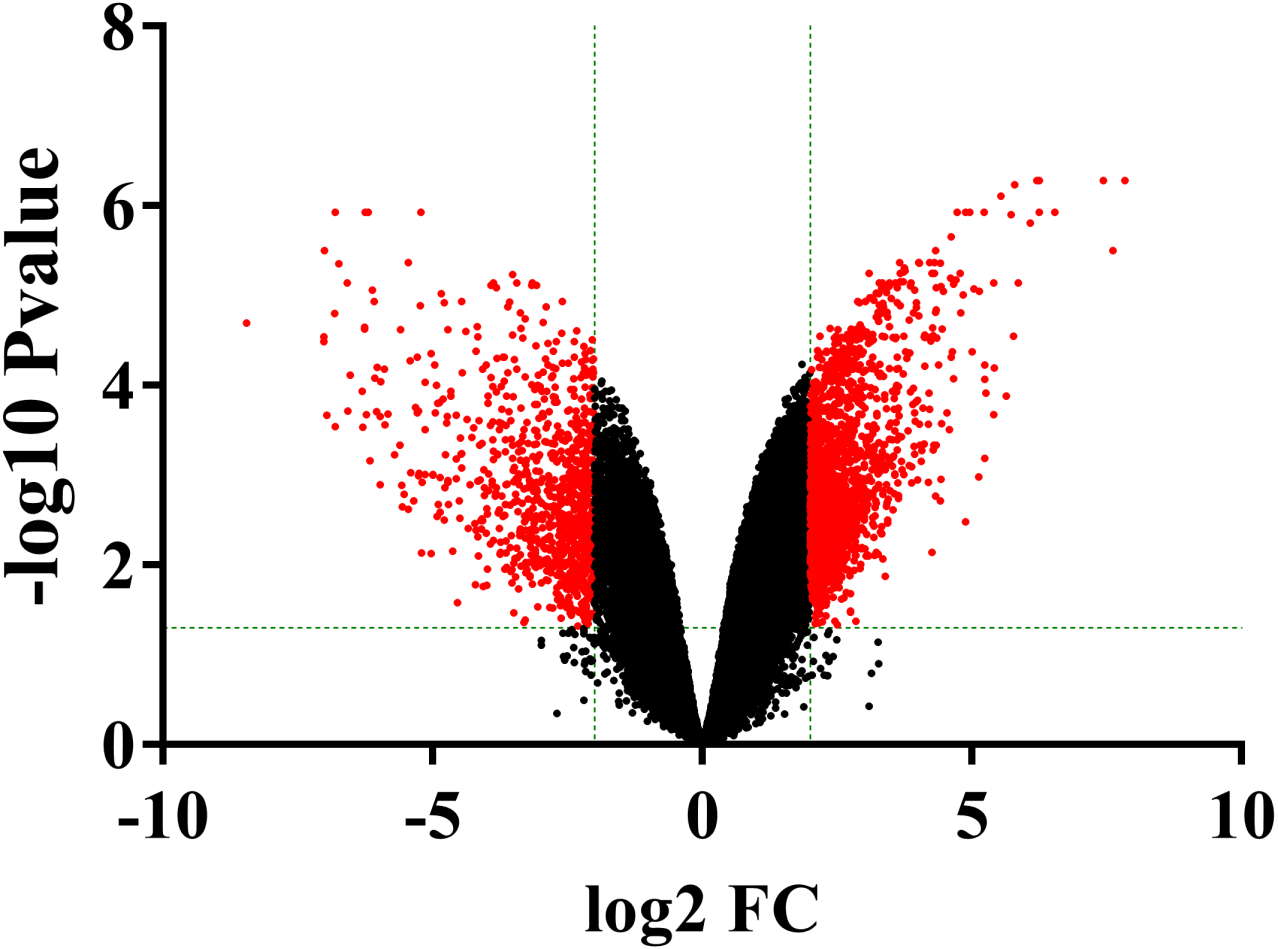
The volcano plot was constructed using fold-change (LogFC) values and adjusted *P*-values. The vertical green lines correspond to 2.0-LogFC up and down and the horizontal green line represents an adjusted *P*-value of 0.05. The red point represents the differentially expressed genes with statistical significance.

The volcano plots showed the variation of gene expression between ONFH and FHF cartilage samples (Fig. 2). Based on the thresholds of adjusted *P* < 0.05 and |LogFC| ≥ 2, a total of 2174 DEGs were obtained in the ONFH cartilage specimens compared to the control group, including 1482 up-regulated and 692 down-regulated ones.

### GO and KEGG enrichment of DEGs

**Figure 3:**
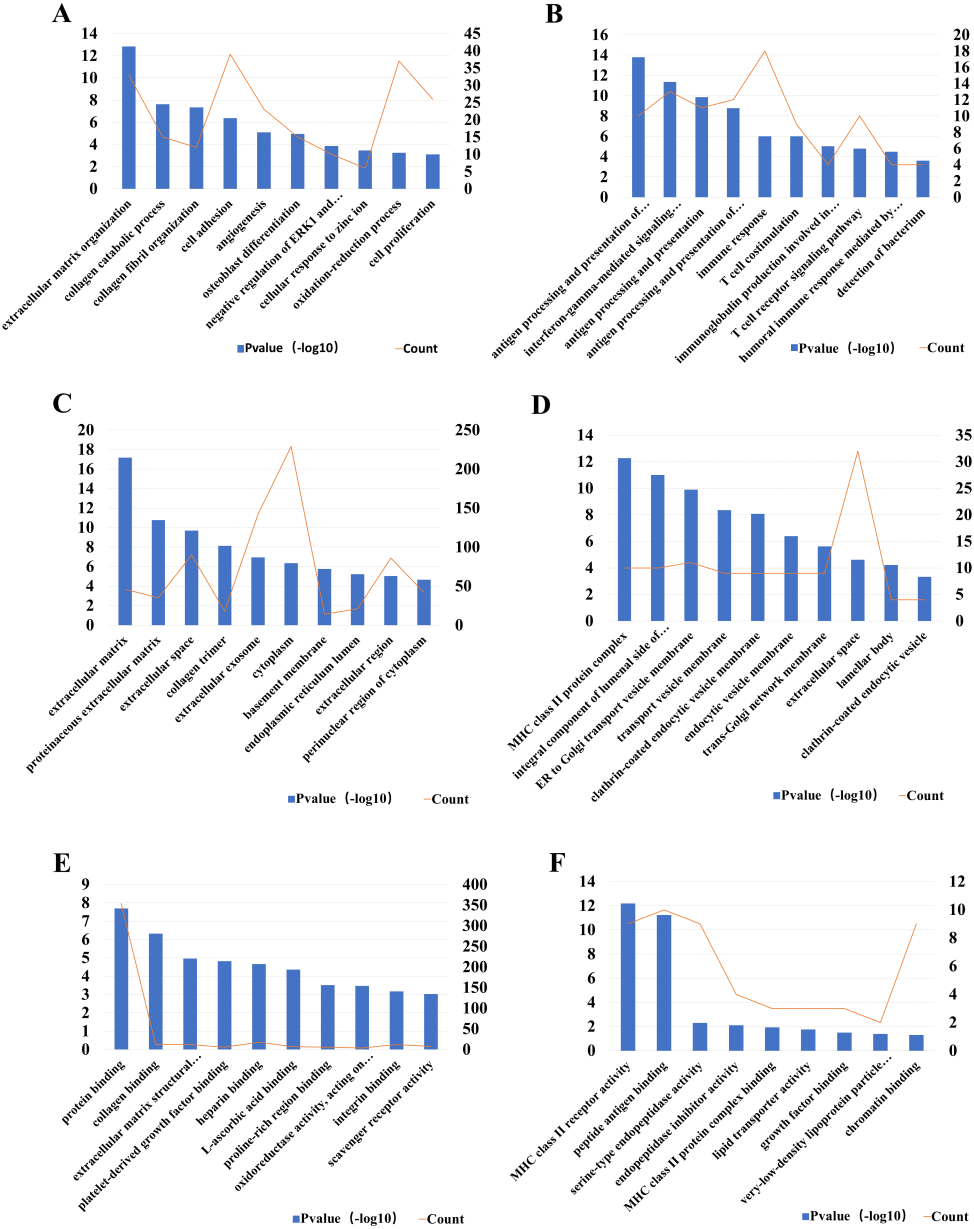
The most 10 significant Gene Ontology (GO) enrichment items and the DEGs count. The biological processes (BP) of (A) up- and (B) down- regulated DEGs, respectively; The cell component (CC) of (C) up- and (D) down-regulated DEGs, respectively; The molecular function (MF) of (E) up- and (F) down-regulated DEGs, respectively. The left *Y*-axes was for *P*-value and the right *Y*-axes was for gene counts.

For a better understanding of the DEGs, GO function and KEGG pathway enrichment analysis were applied using DAVID. The GO analysis results indicated that biological processes (BP) of up-regulated DEGs were particularly enriched in extracellular matrix organization, collagen catabolic process collagen fibril organization and so on. The BP of down-regulated DEGs were mainly enriched in antigen processing and presentation of peptide or polysaccharide antigen via MHC class II, interferon-gamma-mediated signaling pathway, antigen processing, and presentation and so on. For cell component (CC), the upregulated DEGs were enriched in extracellular matrix, proteinaceous extracellular matrix, extracellular space and so on, while the down-regulated DEGs were enriched in MHC class II protein complex, integral component of lumenal side of endoplasmic reticulum membrane, ER to Golgi transport vesicle membrane and so on. The molecular function (MF) analysis showed that the up-regulated DEGs were significantly enriched in the protein binding, collagen binding, and extracellular matrix structural constituent, while down-regulated DEGs enriched in MHC class II receptor activity, peptide antigen binding and serine-type endopeptidase activity (Fig. 3).

KEGG pathway enrichment analysis was also applied by DAVID. Based on the thresholds of *P* < 0.05, the pathway enrichment analysis indicated that the up-regulated DEGs were significantly involved in 18 pathways and the down-regulated DEGs were significantly involved in 23 pathways (*p* < 0.05, FDR-corrected). The five most significant pathways of up-regulated and down-regulated DEGs were listed in Table 4.

**Table 4:** KEGG pathway analysis of differently expressed genes.

Pathway terms	*P*-value	Counts
Up-regulated DEGs		
ECM-receptor interaction	9.86E−09	18
Focal adhesion	2.10E−08	27
PI3K-Akt signaling pathway	1.78E−05	30
Platelet activation	6.68E−05	16
Protein digestion and absorption	3.13E−04	12
Down-regulated DEGs		
Staphylococcus aureus infection	8.10E−20	17
Graft-versus-host disease	8.05E−13	11
Allograft rejection	2.93E−12	11
Type I diabetes mellitus	1.19E−11	11
Asthma	1.34E−11	10

### The expression levels of chondrogenesis-related and arthritis-related genes in cartilage with different Ficat stages

**Figure 4:**
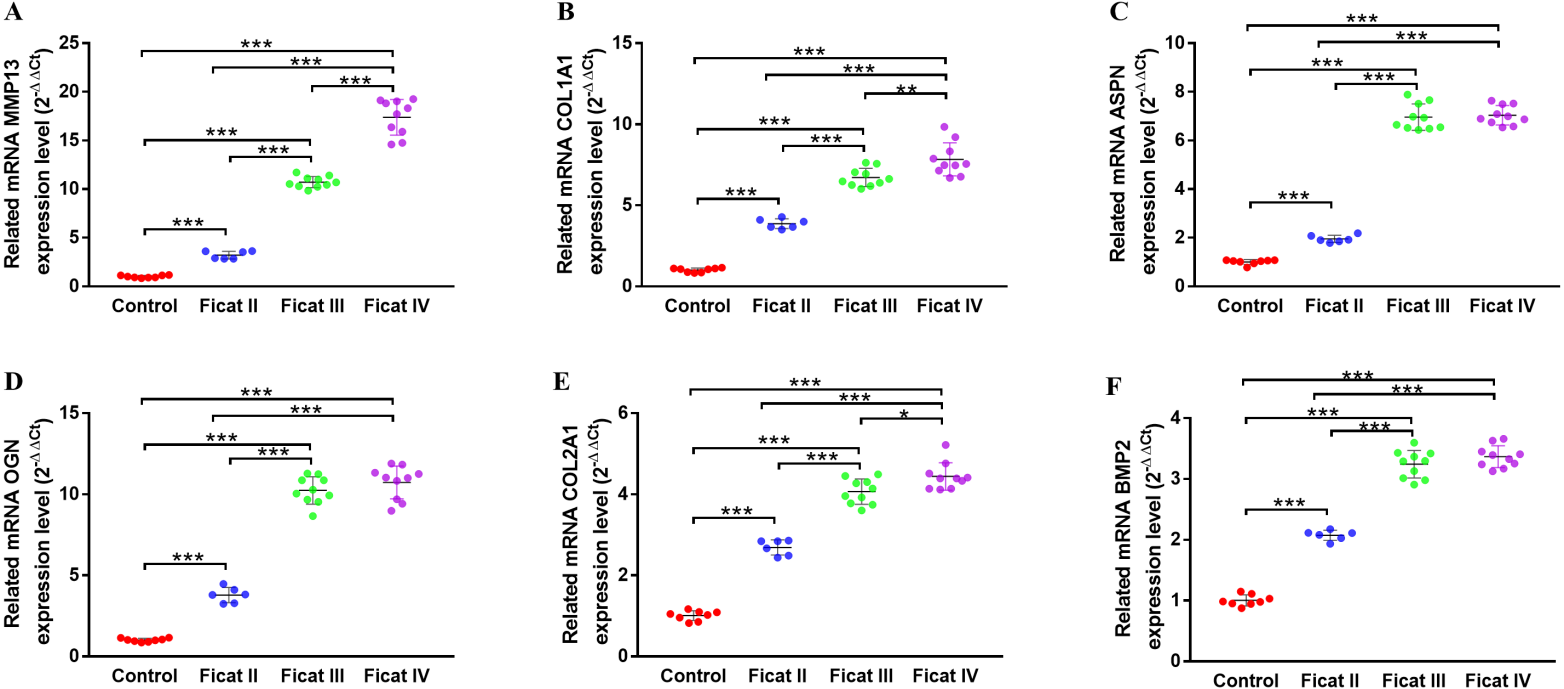
The gene expression levels of chondrogenesis-related genes in cartilage specimens with different Ficat stages. (A) MMP13, (B) ASPN, (C) COL1A1, (D) OGN, (E) COL2A1, (F) BMP2. (*n* = 3; * *p* < 0.05, ** *p* < 0.01, *** *p* < 0.001)

**Figure 5:**
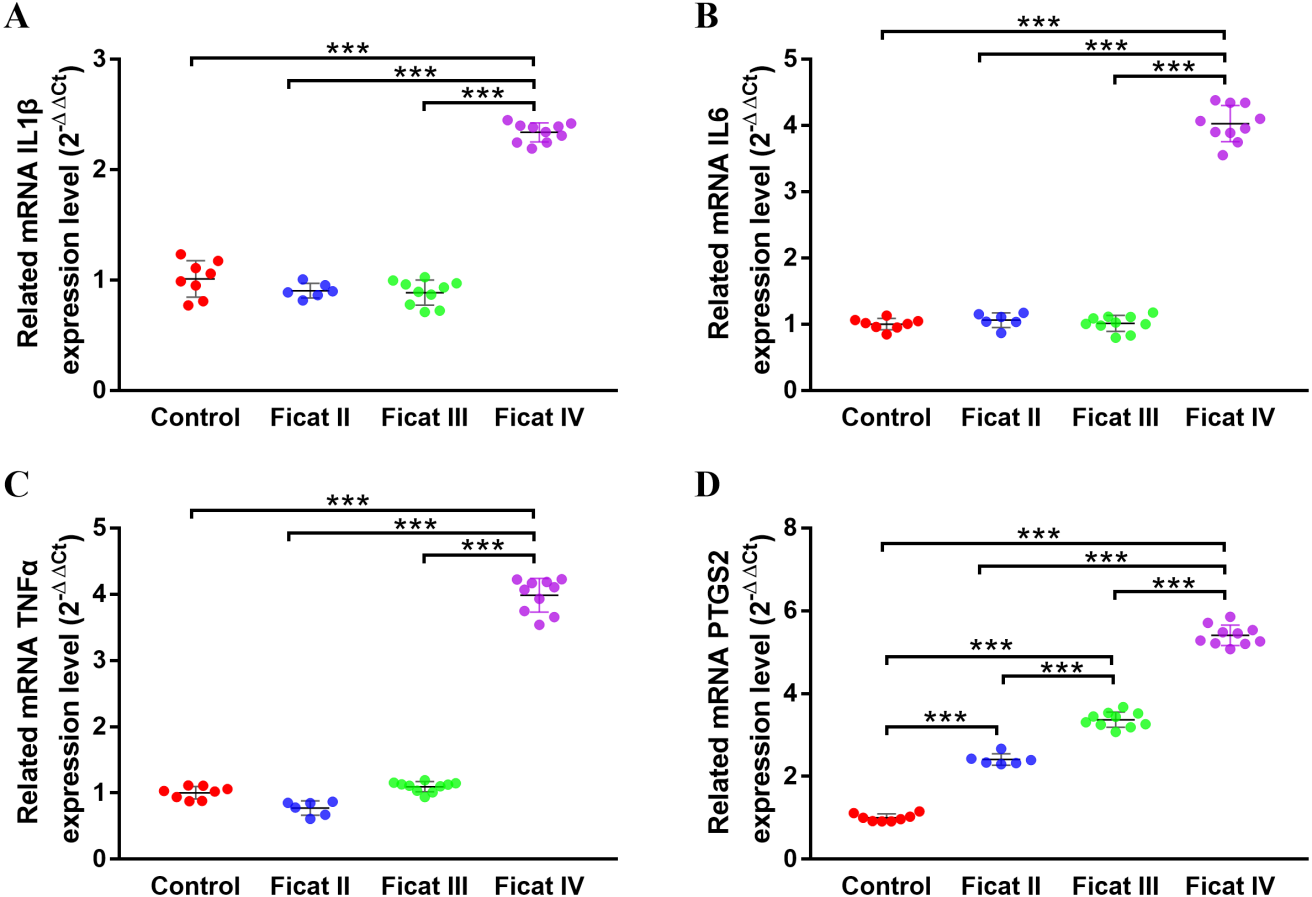
The gene expression levels of arthritis-related genes in cartilage specimens with different Ficat stages. (A) IL1 *β*, (B) IL6 , (C) TNF *α* , D) PTGS2 . (*n* = 3; * *p* < 0.05, ** *p* < 0.01, *** *p* < 0.001).

To further explore the different expression levels of chondrogenesis-related genes and arthritis-related genes in cartilage with different Ficat stages, the qRT-PCR analysis was used to detect the expression levels of six chondrogenesis-related genes (MMP13, ASPN, COL1A1, OGN, COL2A1, BMP2) and four arthritis-related genes (IL1*β*, IL6, TNF*α*, PTGS2) in different Ficat stages of cartilage specimens. The results showed a significant stepwise up-expression of chondrogenesis-related genes and arthritis-related gene PTGS2 along with the progression of ONFH. Other arthritis-related genes IL1*β*, IL6 and TNF*α* were not found differentially expressed in GSE74089, but they were up expressed in the Ficat IV stage (Figs. 4 and 5).

### PPI network of chondrogenesis-related and arthritis-related genes

**Figure 6:**
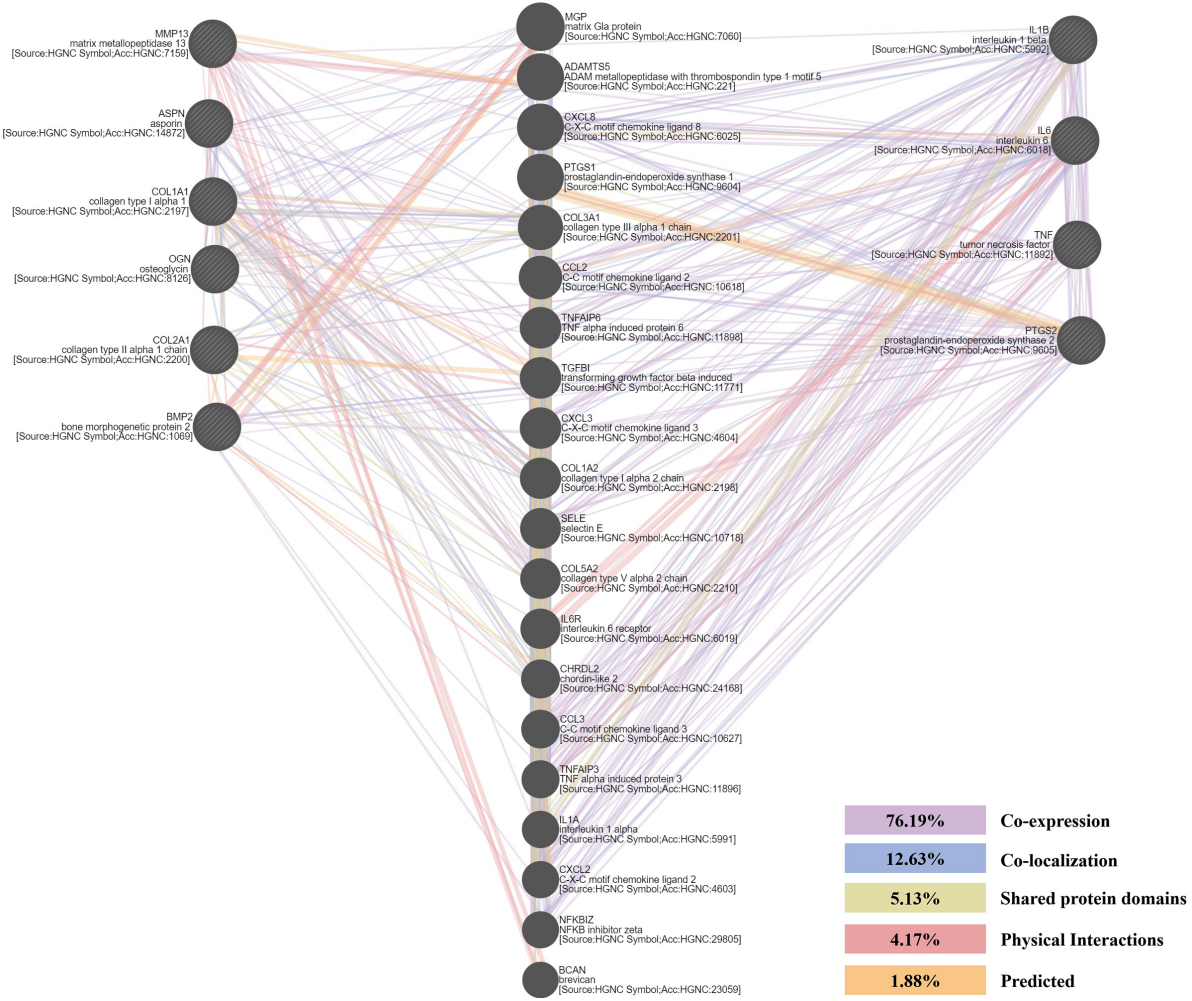
The protein-protein interaction (PPI) network of chondrogenesis-related and arthritis-related genes. Their relationships were presented by lines with different colors. The percentage of co-expression, co-localization, shared protein domains, physical interactions and predicted were 76.19%, 12.63%, 5.13%, 4.17%, 1.88%, respectively.

A PPI network of six chondrogenesis-related and four arthritis-related genes was conducted, based on the information in the GeneMANIA protein query from public databases. Genes related to chondrogenesis-related genes were mainly enriched in cartilage and ECM formation. Genes related to four arthritis-related genes not only enriched in inflammation, but also enriched in growth factors and cartilage formation (Fig. 6).

## Discussion

The degeneration of hip articular cartilage with ONFH has already been characterized by previous studies (Magnussen, Guilak & Vail, 2005; Sonoda et al., 2017). Histologic changes in articular cartilage, including loss of cartilage cells, surface fibrillation, and subchondral bone thickening, may result in differential gene expression in articular cartilage (Im et al., 2000). Liu et al. (2016) conducted genome-wide gene expression profiling of hip articular cartilage in four patients with ONFH (Ficat III grade) and four controls and identified a set of differentially expressed genes in ONFH articular cartilage. We analyzed the gene expression microarray GSE74089 and identified 1482 up-regulated and 692 down-regulated DEGs in ONFH cartilage based on the thresholds of adjusted *P* < 0.05 and |LogFC| ≥ 2. Liu et al. identified 27 differently expressed genes in ONFH articular cartilage in their study. Based on gene function and results from previous study, nine cartilage development and damage related DEGs were selected for qRT-PCR validation in their study, including COL1A1, ANGPTL4, CRTAC1, ASPN, COL3A1, VKORC1, OGN, SPP1 and P4HA2 (Liu et al., 2016). Our results were different to their study as we used different analysis method and thresholds.

For a further understanding of DEGs function, GO and KEGG enrichment analysis was performed via DAVID. The GO and KEGG enrichment items of up-regulated DEGs mainly enriched in extracellular matrix, angiogenesis, osteoblast differentiation, and cell adhesion. However, items of down-regulated mainly enriched in antigen processing and presentation, MHC class II protein complex and peptide antigen binding. These items have been reported to play important role in the pathogenesis of cartilage degeneration in ONFH (Abe, Nochi & Ito, 2016; Ciechomska et al., 2014; García-Fernández, 2018; Lu et al., 2018; Wong et al., 2018). As these results showed, the expression of cartilage and ECM-related gene were up-regulated and induced the process of cartilage rehabilitation in degenerated cartilage, which may result from a number of stimuli factors such as hypoxia and ischemia in cartilage. Simultaneously, the down-regulation of antigen processing associated genes indicated the weakened immune responses in degenerated cartilage, which may be an activation of cartilage protection. The differential expression of these genes may be due to the response to hypoxia and ischemia of cartilage, but it failed to change the fact that the cartilage degenerated progressively.

The DEGs seem to be important not only for their roles in disease progression but also for their potential as molecular markers. In order to further explore the molecular changes along with the progression of ONFH, we selected six chondrogenesis-related and four arthritis-related genes and detected their expression levels in ONFH articular cartilage with different Ficat stages. The results showed a significant stepwise up-expression of chondrogenesis-related genes along with the progression of ONFH (Fig. 4), which indicated that the stimulation of cartilage rehabilitation in degenerated cartilage enhanced with the progress of the disease. To explore the arthritis progress in ONFH, four arthritis-related genes were detected. Although IL1*β*, IL6, and TNF*α* were not found differentially expressed in the Ficat IV stage, they were up expressed in the Ficat IV stage in our verification test, which indicated that the arthritis-related molecular changes were not significant in the progression of ONFH before the Ficat III stage. But there is indeed significant change in the Ficat IV stage. However, PTGS2 was significantly stepwise up-expressed along with the progression of ONFH which makes it to be a sensitive biomarker in the arthritis process of ONFH. In order to explore the interaction and relationship between chondrogenesis-related genes and arthritis-related genes, we conducted a PPI network and the result showed their relationships mainly enriched in co-expression (76.19%) and co-location (12.63%). The PPI result indicated that there are many interconnections among these genes which may play critical roles in the progression of ONFH.

It should be noted that there are still some limitations in our study. Large sample size was still needed to confirm our findings. In addition, further studies are needed to explore the exact regulation mechanism between chondrogenesis-related genes and arthritis-related genes in the progression of ONFH.

## Conclusion

We analyzed a gene expression profile of ONFH and validated the expression changes of selected six chondrogenesis-related and four arthritis-related genes in hip articular cartilage specimens with different ONFH Ficat stages. These findings are expected to get a further insight into the molecular mechanisms of ONFH progression.

##  Supplemental Information

10.7717/peerj.6306/supp-1 Data S1 Microarray raw data

## Additional Information and Declarations

### Data Availability

The following information was supplied regarding data availability: NCBI GEO number GSE74089.
